# Evidence-based Birth Attendance in Spain: Private *versus* Public Centers

**DOI:** 10.3390/ijerph16050894

**Published:** 2019-03-12

**Authors:** Antonio Hernández-Martínez, Juan Miguel Martínez-Galiano, Julián Rodríguez-Almagro, Miguel Delgado-Rodríguez, Juan Gómez-Salgado

**Affiliations:** 1 Mancha-Centro Hospital, Alcázar de San Juan, 13600 Ciudad Real, Spain; antomatron@gmail.com; 2 Department of Nursing, University of Jaén, Campus de Las Lagunillas s/n, Building B3 Office 266, 23071 Jaén, Spain; jgaliano@ujaen.es; 3 Consortium for Biomedical Research in Epidemiology and Public Health (CIBERESP), 28029 Madrid, Spain; mdelgado@ujaen.es; 4 Department of Nursing, University of Castilla la Mancha, 13071 Ciudad Real, Spain; 5 Division of Preventive Medicine and Public Health, University of Jaen, 23071 Jaen, Spain; 6 Department of Nursing, Faculty of Nursing, University of Huelva, 21071 Huelva, Spain; jgsalgad@gmail.com; 7 Espíritu Santo University, Guayaquil 092301, Ecuador

**Keywords:** birth, maternal outcomes, public hospital, private hospital, care suppliers

## Abstract

The type of hospital (public or private) has been associated with the type of clinical practice carried out. The purpose of this study was to determine the association between the type of hospital (public or private) and delivery attendance with practices based on the recommendations by the World Health Organization (WHO). A cross-sectional study with puerperal women (*n* = 2906) was conducted in Spain during 2017. The crude Odds Ratios (OR), adjusted (aOR) and their 95% confidence intervals (CI) were calculated through binary logistic regression. For multiparous women in private centers, a higher rate of induced labor was observed (aOR: 1.49; 95% CI: 1.11–2.00), fewer natural methods were used to relieve pain (aOR: 0.51; 95% CI: 0.35–0.73), and increased odds of cesarean section (aOR: 2.50; 95% CI: 1.81–3.46) were found as compared to public hospitals. For primiparous women in private centers, a greater use of the epidural was observed (aOR: 1.57; 95% CI: 1.03–1.40), as well as an increased likelihood of instrumental birth (aOR: 1.53; 95% CI: 1.09–2.15) and of cesarean section (aOR: 1.77; 95% CI: 1.33–2.37) than in public hospitals. No differences were found in hospitalization times among women giving birth in public and private centers (*p* > 0.05). The World Health Organization birth attendance recommendations are more strictly followed in public hospitals than in private settings.

## 1. Introduction

The World Health Organization (WHO), in the Fortaleza Declaration, provides birth attendance recommendations to promote non-medicalized assistance without interventions [1]. To a great extent, these recommendations have been implemented by public health administrations in many countries worldwide via different plans, strategies and guidelines [2,3,4]. Moreover, scientific societies engaged in perinatal assistance have developed clinical practice guidelines and evidence-based action protocols that are available online. The WHO frames these actions within the process of birth attendance [5,6,7].

The main care supplier regarding birth attendance in most health systems worldwide is the public health administration [8,9,10,11,12,13]. Nevertheless, private companies are the main providers of birth attendance in some countries like Singapore [14] and USA [15]. Despite the public health system dominating this background, some countries are presently witnessing a growing trend towards the provision of care by private suppliers instead of the public health system [16,17,18].

To date, different studies have associated the type of center with the skilled birth attendance recommendations by the WHO, based on the private or public character of centers [19,20,21,22,23,24]. A retrospective cohort study conducted in Ireland with 403,642 women detected that those women with private health policies were are at a higher risk of having a cesarean section, either by choice (relative risk: RR: 1.48; 95% CI: 1.45–1.51) or as an emergency measure (RR: 1.13; 95% CI: 1.12–1.16), as well as a higher probability of having an instrumental birth (RR: 1.25; 95% CI: 1.22–1.27) or an episiotomy (RR: 1.40; 95% CI: 1.38–1.43), as compared to those women who give birth in public hospitals [19]. A systematic review of 18 articles showed a higher risk of having a cesarean section in private hospitals than in public ones (OR: 1.35; 95% CI: 1.27–1.44) [20]. However, another study conducted on 617,269 births found no association between the type of hospital (public or private) and cesarean sections (OR: 1.01; 95% CI: 0.99–1.02) [21]. In Thailand, the results of a study on 11,049 women proved a higher risk of giving birth by cesarean in private hospitals than in public ones (OR: 9.44; 95% CI: 8372–10,655) [22].

Despite three decades having elapsed since the WHO made its birth attendance recommendations, which are mainly aimed at reducing the rate of cesareans, among other objectives, existing studies reveal the reluctance of private hospitals to include them in their clinical practice. In recent years, a twofold trend has been observed: on the one hand, scientific evidence supports more humanized birth attendance while, on the other hand, more women choose a private hospital to give birth. The aim of this study was to identify whether there is an association between the type of hospital where the woman gives birth (public or private) and the performance of the clinical practices recommended by the WHO on attendance at delivery.

## 2. Materials and Methods

A cross-sectional study was conducted with a total sample of 3437 women who gave birth in Spain in 2017.

The inclusion criterion was singleton term pregnancies. The exclusion criteria included pregnancies that ended in antenatal fetal death, giving birth at home, breech delivery, and those women whose birth was initially attended to at home and ended in a hospital.

We estimated the appropriate sample size according to the results of a similar study [2]. The likelihood of a cesarean section was considered the main variable, and the cesarean rate in private centers was used as a reference (35%), along with the cesarean rate in public centers (23%) [2]. To detect a significant 5% alpha risk and a 10% beta risk (power = 90%), a minimum of 298 women per group was estimated.

**Figure 1:**
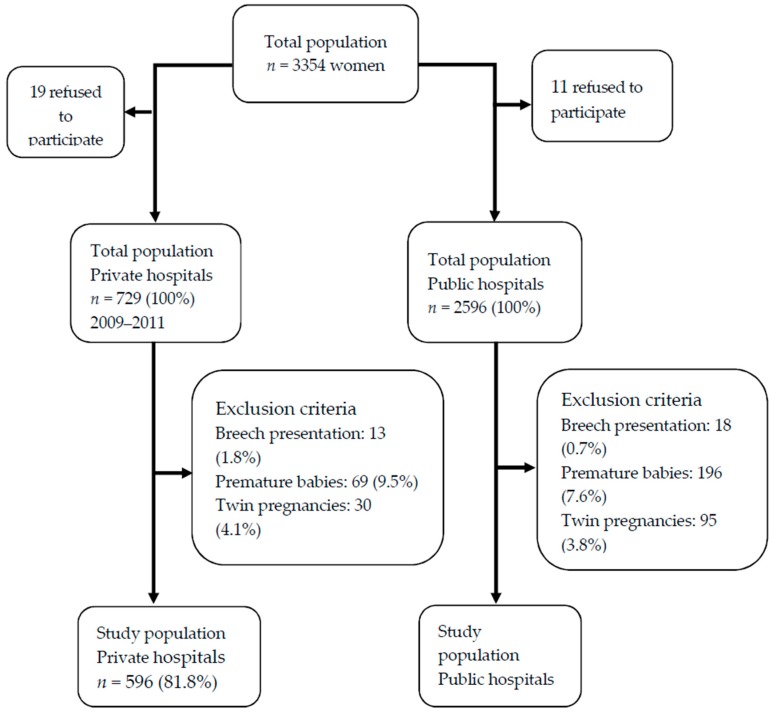
Diagram showing the process followed for the selection of the study subjects.

As this is an observational study, in order to know the prevalence of birth attendance in both types of centers, the recruitment of women for the study was not restricted to a given number. Instead, the recruitment time was limited to three months, that is, the data collection was carried out during three months, and all the women who gave birth in that period of time and who met the inclusion criteria and wished to participate were included. Figure 1 shows a diagram of the process followed for the selection of participants.

### 2.1. Information Sources

An online questionnaire devised by the authors was used for data collection. It comprised 35 items (three open and 32 closed questions) about socio-demographic and clinical characteristics, obstetric results, and data on newborns. The questionnaire was previously trialed and distributed to women via the main women associations, the Spanish Federation of Midwives Associations (FAME) and its member associations that engaged midwives in the dissemination of the project and encourage women to participate. After the study subjects’ selection and upon their acceptance to participate, the midwives in charge of recruiting the women provided them with instructions to fill in the questionnaire, which the final participants did according to their availability. Telephone assistance and a scheduled chat were provided to clarify any doubts these women may have when filling the questionnaire.

### 2.2. The Following Variables were Collected

The main independent variable was the type of center where birth attendance was provided (private/public). The main outcome variables were: induced birth, preparing a birth plan, employing local analgesia (epidural/rachianesthesia), employing natural methods to relieve pain, application of fundal pressure during the second stage of labor (Kristeller maneuver), cesarean, re-hospitalization after hospital discharge, postnatal surgery, mother admitted to an intensive care unit (ICU), skin-to-skin contact at birth, newborn admitted to hospital, feeding/milk type, and length of hospital stay. The following were also selectively evaluated as outcomes for women who gave birth vaginally: birth type (instrumental/eutocic), practicing episiotomy, and presence of severe tearing (grade III or IV).

The secondary variables taken into account to control confounding factors were of a socio-demographic and clinical type. In addition, the variables that could potentially act as confounders were used for each outcome.

### 2.3. Statistical Analysis

First, a descriptive analysis was carried out for which absolute and relative frequencies were used. Then, a bivariate analysis between the type of center and the main obstetric and neonatal results was done by stratifying by parity (primiparous/multiparous): the odds ratios (OR) were calculated, along with the 95% confidence interval (95% CI) and the Pearson’s chi-squared test. Then, a multivariate analysis was performed by means of binary logistic regression, which was stratified by parity. In addition, the potential confounding variables were used for each analysis. Finally, both types of center were compared regarding the length of hospital stay by stratifying by birth type, for which a non-parametric Mann-Whitney *U* test was used. A *p* ˂ 0.05 was considered significant. Additionally, all the analyses were done using the SPSS v24.0 (SPSS Inc., Chicago, IL, USA) statistical package.

### 2.4. Ethic-Legal Considerations

This study was approved by the Ethical Committee on Clinical Research (CEIC, for its Spanish acronym) with ethical code 69-C of the La Mancha-Centro Centre. Before starting the questionnaire, the participating women read a fact sheet about the study, its objectives, etc., and marked a box by which they showed their consent to participate in it, i.e., they signed an online informed consent (ticking the option if they wanted to participate or not doing so when refusing to take part in the study).

## 3. Results

2906 women were recruited, of whom 596 attended private hospitals and 2310 public ones. Therefore, public hospitals received 79.5% of the study women and 20.5% of them attended private ones. Table 1 provides the population characteristics, where we find older age and a higher level of education, among others, as the factors associated with giving birth in private centers (*p* < 0.001). In addition, multiparous women giving birth in public centers were associated with attendance to antenatal childbirth classes (*p* < 0.001) and the presence of health problems during pregnancy (*p* = 0.043).

Table 2 shows an inverse association between primiparous women who gave birth in private hospitals and performing an episiotomy (aOR: 0.67; 95% CI: 0.47–0.96).

Those primiparous women who gave birth in private hospitals were also associated with a more frequent use of epidural (aOR: 1.57; 95% CI: 1.03–1.40) than their counterparts in public hospitals. Moreover, primiparous women who gave birth in private hospitals were associated with a greater likelihood of having an instrumental birth (aOR: 1.53; 95% CI: 1.09–2.15) or cesarean section (aOR: 1.77; 95% CI: 1.33–2.37), as opposed to those who gave birth in public hospitals.

Regarding multiparous women who attended private centers, we observed a significantly higher rate of induced births (aOR: 1.49; 95% CI: 1.11–2.00), a greater use of analgesia (aOR: 2.58; 95% CI: 1.83–3.63), fewer natural methods to ease pain (aOR: 0.51; 95% CI: 0.35–0.73), and more births that ended in cesarean (aOR: 2.50; 95% CI: 1.81–3.46) (Table 3).

As Table 4 shows, the newborns born to primiparous women who were assisted in private hospitals were positively associated with early skin-to-skin contact at birth (aOR: 1.65; 95% CI: 1.17–2.34). No association was found between public or private hospitals and length of postnatal hospital stay for primiparous women, nor for the birth being eutocic (*p* = 0.226), instrumental (*p* = 0.988), or ending in a cesarean section (*p* = 0.101).

## 4. Discussion

According to the results found in this study, birth attendance in private hospitals entailed more cesareans for both primiparous and multiparous women, and with a greater use of epidural analgesia. More primiparous women had instrumental births in private hospitals, but fewer women underwent episiotomies. In addition, skin-to-skin contact with their babies took place earlier than in public hospitals. More multiparous women births in private centers started in an unspontaneous way, and the professional staff resorted to fewer natural methods to relieve pain than in public centers. No differences were found between public and private hospitals regarding the length of post-birth hospital stay as for any birth type.

It was decided to exclude premature newborns in order to achieve standardized comparisons between the centers. The incidence of premature newborns is highly variable depending on the level of care of the centers. Several limitations are identified in this study. A priori, it is worth thinking that if a selection bias exists and is associated with not participating in the study, this has not influenced the results, as the majority of women agreed to participate and only 30 refused doing so. Nor was there any reason to believe that those who did not participate would have had performed differently to those who did. It is unlikely that an information bias exists as the collected data and the way the possible answers were presented did not require having a high level of education. In this respect, the questions were set out in a basic and simple way, and could be understood by all the participants regardless of their level of education. On the other hand, it is not possible to completely rule out an anamnesis bias. Despite the information being collected over a short time interval, we believe that this would have minimally influenced the results. Moreover, women perfectly knew their health supplier and were able to indicate if they had been assisted in a private or public hospital. They also accurately remembered any information regarding their birth process, which was accordingly evaluated by the vast majority and regarded as a relevant process to devote attention to. Likewise, it is not possible to completely rule out the confounding bias inherent to observational studies. However, we believe that its effect on the study results was not significant as it was considered and controlled during the study design. This was done by using inclusion and exclusion criteria, as well as a multivariate analysis adjusted by confounding factors (found in the scientific literature and in clinical practice experiences) that could have influenced the results. Another limitation is that the data used in the study were of an individual nature for each participant, so it cannot be assured that these outcomes act as indicators and results per center. In other words, it was not possible to find private centers with better results than public ones. However, the present study analyzed them overall.

More women who gave birth in private centers had a cesarean section than those who gave birth in public hospitals, regardless of being primiparous or multiparous. This finding agrees with other authors’ results [14,19,20,22]. Nevertheless, in a study carried out on 617,269 live-birth deliveries in Michigan (USA), no association between public or private hospitals and cesarean births was found [21]. A recent cross-sectional study conducted on 323 women in Australia associated private hospitals with a higher risk of cesarean [25]. In the results of the present study, the presence of a pathology during pregnancy was associated to giving birth in a public hospital regarding multiparous women (possible clinical criteria indicating cesarean) and would have determined higher cesarean rates in the public centers. Despite this, the cesarean rate among multiparous women was higher in private centers, so, a priori, this clinical criterion was not decisive for the cesarean rate in multiparous women. However, a difference was found regarding the women’s level of education and age and the choice to give birth in private centers. These outcomes have been identified as risk factors for giving birth by cesarean by Suarez-Lopez et al. [26]. Also, in line with the present study results, this greater likelihood of giving birth by cesarean in private hospitals, according to several authors, may be due to non-clinical variables such as social factors (age, level of education, etc.) and economic ones [27,28].

On the one hand, our results also revealed that multiparous women who decided to give birth in private hospitals were more likely to have an induced birth, which contrasts with the results obtained by Wilkes et al. [25]. On the other hand, the present study is in line with the outcomes reported in a study carried out on all maternities in France [29]. According to our results, induced births are more likely to happen in private hospitals. This could also be a determining factor to have a cesarean section in private centers due to the fact that induced labor has been identified as a risk factor for a cesarean section [29].

During the study process, a greater likelihood of instrumental birth was found for primiparous women in private centers, along with regional analgesia being more frequently used (epidural/ rachianesthesia). These results coincide with other authors’ outcomes [17,19,30,31]. In line with our conclusions, and in addition to epidural being more frequently used, a Brazilian study has shown an association between giving birth in private hospitals and not using alternative analgesic methods during birth [30].

Primiparous women giving birth in private hospitals came over as a protective factor against performing episiotomy, unlike the results obtained by Escuriet et al. in their study conducted in Spain [32] and those reported by other authors [17,19,30,31]. The cause of this higher rate of episiotomies may be due to the fact that there are more vaginal births in public hospitals than in private ones, as private centers have a higher rate of cesarean sections.

Earlier skin-to-skin contact was also identified in women giving birth in private hospitals than in those who did it in public ones, but no influence was detected on any other studied practice or intervention: moment of starting breastfeeding, newborn being admitted to hospital, presence of severe perineal tearing, mother being admitted to an ICU, length of hospital stay, mother being readmitted to hospital, performing Kristeller maneuver during expulsive contractions, performing surgery, and women using a birth plan. Some of the analyzed practices, e.g., using a birth plan, have been studied by Prado et al. [30], with results in line with those found in this study.

Private centers policies should include the recommendations by the WHO on birth attendance as a mandatory standard to be followed by the professionals working in these hospitals. In addition, the staff should be trained in and sensitized on the importance of carrying out childbirth attendance based on the WHO recommendations.

## 5. Conclusions

To conclude, clinical practices regarding birth attendance in public hospitals do follow the WHO recommendations and the available scientific evidence to a greater extent than private hospitals. Thus, giving birth in a private center is associated with an increased likelihood of delivery by cesarean and use of epidural analgesia during delivery. In addition, primiparous women have been associated with a greater number of instrumental deliveries and less early skin-to-skin contact with the newborn. Finally, multiparous women show a higher incidence of induced labor.

## Figures and Tables

**Table 1:** Study population’s characteristics.

Variable	Primiparous			Multiparous		
	Hospital Type		*p*	Hospital Type		*p*
	Private *n* (%) 274	Public *n* (%) 1223		Private *n* (%) 322	Public *n* (%) 1087	
Mother’s age			**0.007**			**0.001**
<35 years	145 (52.9)	755 (61.7)		110 (43.2)	480 (44.2)	
≥35 years	129 (47.1)	468 (38.3)		212 (65.8)	607 (55.8)	
Level of education			**0.010**			**<0.001**
No qualifications	0 (0.0)	5 (0.4)		0 (0.0)	2 (0.2)	
Primary education	4 (1.5)	48 (3.9)		6 (1.9)	75 (6.9)	
Secondary education	85 (31.0)	462 (37.8)		94 (29.2)	415 (38.2)	
University education	185 (67.5)	708 (57.9)		222 (68.9)	595 (54.7)	
Attendance to antenatal classes			0.802			**<0.001**
No	22 (8.0)	112 (9.2)		173 (53.7)	429 (39.5)	
Yes, but fewer than five classes	37 (13.5)	155 (12.7)		41 (12.7)	143 (13.2)	
Yes, a minimum of five classes	215 (78.5)	956 (78.2)		108 (33.5)	515 (47.4)	
Nationality			0.680			0.760
Spanish	262 (95.6)	1176 (96.2)		313 (97.2)	1053 (96.9)	
Other	12 (4.4)	47 (3.8)		9 (2.8)	34 (3.1)	
Health problems during pregnancy			0.812			**0.043**
No	200 (73.0)	884 (72.3)		243 (75.5)	757 (69.6)	
Yes	74 (27.0)	339 (27.3)		79 (24.5)	330 (30.4)	
Previous cesarean			NA			0.140
No	274 (100)	1223 (100)		276 (85.7)	947 (87.1)	
Yes	NA	NA		46 (14.3)	140 (12.9)	

NA: Not applicable; Bold: Significant results are highlighted.

**Table 2:** Association between the type of hospital (public or private) where delivery occurs and the probability of some clinical practices in primiparous women.

Variable	Hospital		OR 95% CI	aOR 95% CI
	Private *n* (%)	Public *n* (%)		
Induced birth				
No	162 (59.1)	752 (61.5)	1 (ref.)	1 (ref.) ^a^
Yes	112 (40.9)	471 (38.5)	1.11 (0.85–1.44)	1.05 (0.78–1.42)
Birth plan				
No	218 (79.6)	954 (77.9)	1 (ref.)	1 (ref.) ^b^
Yes	56 (20.4)	270 (22.1)	0.91 (0.66–1.25)	0.90 (0.65–1.24)
Use of epidural/rachianesthesia				
No	29 (10.6)	194 (15.9)	1 (ref.)	1 (ref.) ^c^
Yes	245 (89.4)	1029 (84.1)	**1.59 (1.05–2.41)**	**1.57 (1.03–1.40)**
Use of natural methods to ease pain				
No	197 (71.9)	822 (67.3)	1 (ref.)	1 (ref.) ^d^
Yes	77 (28.1)	399 (32.7)	0.81 (0.60–1.08)	0.79 (0.58–1.07)
Kristeller maneuver				
No	148 (54.0)	733 (59.9)	1 (ref.)	1 (ref.) ^d^
Yes	126 (46.0)	490 (40.1)	1.11 (0.85–1.44)	1.27 (0.97–1.66)
Type of vaginal birth				
Eutocic/normal	96 (55.2)	617 (66.6)	1 (ref.)	1 (ref.) ^e^
Instrumental	78 (44.8)	310 (33.4)	**1.62 (1.17–2.25)**	**1.53 (1.09–2.15)**
Cesarean				
No	174 (63.5)	927 (75.8)	1 (ref.)	1 (ref.) ^f^
Yes	100 (36.5)	296 (24.2)	**1.80 (1.36–2.38)**	**1.77 (1.33–2.37)**
Severe tearing (grade III/IV)				
No	166 (95.4)	854 (92.1)	1 (ref.)	1 (ref.) ^g^
Yes	8 (4.6)	73 (7.9)	0.56 (0.27–1.19)	0.49 (0.23–1.05)
Episiotomy				
No	76 (43.7)	362 (39.1)	1 (ref.)	1 (ref.) ^g^
Yes	98 (56.3)	565 (60.9)	0.83 (0.60–1.15)	**0.67 (0.47–0.96)**
Mother admitted to an ICU				
No	271 (98.9)	1202 (98.3)	1 (ref.)	1 (ref.) ^h^
Yes	3 (1.1)	21 (1.7)	0.63 (1.89–2.14)	0.44 (0.13–1.52)
Post-birth surgery				
No	268 (97.8)	1188 (97.1)	1 (ref.)	1 (ref.) ^h^
Yes	6 (2.2)	35 (2.9)	0.76 (0.32–1.83)	0.74 (0.31–1.80)
Readmitted to hospital after discharge from hospital				
No	266 (97.1)	1181 (96.6)	1 (ref.)	1 (ref.) ^h^
Yes	8 (2.9)	42 (3.4)	0.85 (0.39–3.52)	0.84 (0.34–1.82)

^a^ Adjusted by: age, problems during pregnancy and birth plan. ^b^ Adjusted by: age, problems during pregnancy, induction, nationality, level of education and attending antenatal classes. ^c^ Adjusted by: age, problems during pregnancy, induction, nationality, level of education, attending antenatal classes and birth plan. ^d^ Adjusted by: age, problems during pregnancy, induction, level of education, attending antenatal classes and birth plan. ^e^ Adjusted by: age, induction, problems during pregnancy, using epidural and macrosomy. ^f^ Adjusted by: age, induction, problems during and macrosomy. ^g^ Adjusted by: age, induction, problems during pregnancy, using epidural and macrosomy. ^h^ Adjusted by: age, problems during pregnancy, induction and birth type. Bold: Significant results are highlighted.

**Table 3:** Association between the type of hospital (public or private) where delivery occurs and the probability of some clinical practices in multiparous women.

Variable	Hospital		OR 95% CI	aOR 95% CI
	Private *n* (%)	Public *n* (%)		
Induced birth				
No	215 (66.8)	807 (74.2)	1 (ref.)	1 (ref.) ^a^
Yes	107 (33.2)	280 (25.8)	**1.44 (1.06–1.88)**	**1.49 (1.11–2.00)**
Birth plan				
No	265 (82.3)	856 (78.7)	1 (ref.)	1 (ref.) ^b^
Yes	57 (17.7)	231 (21.3)	0.80 (0.58–1.10)	0.81 (0.58–1.13)
Use of epidural/rachianesthesia				
No	52 (16.1)	389 (35.8)	1 (ref.)	1 (ref.) ^c^
Yes	270 (83.9)	697 (64.2)	**2.89 (2.10–3.99)**	**2.58 (1.83–3.63)**
Use of natural methods to ease pain				
No	271 (84.2)	799 (73.5)	1 (ref.)	1 (ref.) ^d^
Yes	51 (15.8)	288 (26.5)	**0.52 (1.38–0.73)**	**0.51 (0.35–0.73)**
Kristeller maneuver				
No	239 (74.2)	859 (79.0)	1 (ref.)	1 (ref.) ^d^
Yes	83 (25.8)	228 (21.0)	1.31 (0.98–1.75)	1.20 (0.89–1.62)
Type of vaginal birth				
Eutocic/normal	197 (82.1)	831 (87.1)	1 (ref.)	1 (ref.) ^e^
Instrumental	43 (17.9)	123 (12.9)	**1.48 (1.01–2.16)**	1.19 (0.78–1.80)
Cesarean				
No	240 (74.5)	954 (87.8)	1 (ref.)	1 (ref.) ^f^
Yes	82 (25.5)	133 (12.2)	**2.45 (1.80–3.34)**	**2.50 (1.81–3.46)**
Severe tearing (grade III/IV)				
No	232 (96.7)	918 (96.2)	1 (ref.)	1 (ref.) ^g^
Yes	8 (3.3)	36 (3.8)	0.75 (0.88–1.92)	0.71 (0.32–1.60)
Episiotomy				
No	126 (52.5)	593 (62.2)	1 (ref.)	1 (ref.) ^g^
Yes	114 (47.5)	361 (37.8)	**1.49 (1.12–1.98)**	1.26 (0.92–1.72)
Mother admitted to an ICU				
No	318 (98.8)	1075 (98.9)	1 (ref.)	1 (ref.) ^h^
Yes	4 (1.2)	12 (1.1)	1.13 (0.36–3.52)	0.77 (0.24–2.47)
Post-birth surgery				
No	314 (97.5)	1069 (98.3)	1 (ref.)	1 (ref.) ^h^
Yes	8 (2.5)	18 (1.7)	1.51 (0.65–3.52)	1.19 (0.50–2.81)
Readmitted to hospital after discharge from hospital				
No	316 (98.1)	1055 (97.1)	1 (ref.)	1 (ref.) ^h^
Yes	6 (1.9)	32 (2.9)	0.63 (0.26–1.51)	0.52 (0.21–1.27)

^a^ Adjusted by: age, previous cesarean, problems during pregnancy and birth plan. ^b^ Adjusted by: age, previous cesarean, problems during pregnancy, induction, nationality, level of education and attending antenatal classes. ^c^ Adjusted by: age, previous cesarean, problems during pregnancy, induction, nationality, level of education, attending antenatal classes and birth plan. ^d^ Adjusted by: age, previous cesarean, problems during pregnancy, induction, level of education, attending antenatal classes and birth plan. ^e^ Adjusted by: age, previous cesarean, induction, problems during pregnancy, using epidural and macrosomy. ^f^ Adjusted by: age, previous cesarean, induction, problems during and macrosomy. ^g^ Adjusted by: age, induction, problems during pregnancy, using epidural and macrosomy. ^h^ Adjusted by: age, previous cesarean, problems during pregnancy, induction and birth type.

**Table 4:** Association between the type of hospital (public or private) where delivery occurs and the probability of some clinical practices in the newborn.

Variable	Hospital Type		OR 95% CI	aOR 95% CI
	Private *n* (%)	Public *n* (%)		
Primiparous				
Skin-to-skin contact				
No	96 (35.0)	418 (34.2)	1 (ref.)	1 (ref.) ^a^
Yes	178 (65.0)	805 (65.8)	0.96 (0.73–1.27)	**1.65 (1.17–2.34)**
Newborn admitted to hospital				
No	257 (93.8)	1120 (91.6)	1 (ref.)	1 (ref.) ^b^
Yes	17 (6.2)	103 (8.4)	0.72 (0.42–1.22)	0.69 (0.40–1.18)
Artificial feeding/milk				
No	182 (66.4)	821 (67.1)	1 (ref.)	1 (ref.) ^c^
Yes	92 (33.6)	402 (32.9)	1.03 (0.78–1.36)	0.99 (0.75–1.34)
Multiparous				
Skin-to-skin contact				
No	107 (33.2)	223 (20.5)	1 (ref.)	1 (ref.) ^a^
Yes	215 (66.8)	864 (79.5)	**0.52 (0.39–0.68)**	0.75 (0.54–1.05)
Newborn admitted to hospital				
No	308 (95.7)	1022 (94.0)	1 (ref.)	1 (ref.) ^b^
Yes	14 (4.3)	65 (6.0)	0.72 (0.40–1.29)	0.59 (0.32–1.09)
Artificial feeding/milk				
No	260 (80.7)	876 (80.6)	1 (ref.)	1 (ref.) ^c^
Yes	62 (19.3)	211 (19.4)	1.03 (0.78–1.36)	0.95 (0.68–1.33)

^a^ Adjusted by: age, birth type, problems during pregnancy, induction, level of education, attending antenatal classes and birth plan. ^b^ Adjusted by: age, birth type, problems during pregnancy and induction. ^c^ Adjusted by: age, birth type, problems during pregnancy, induction, level of education, attending antenatal classes, birth planned, skin-to-skin contact.
